# Nominal material density may not reflect TPS‐relevant density for 3D‐printed surgical reconstruction implants: An educational case study

**DOI:** 10.1002/acm2.70716

**Published:** 2026-07-26

**Authors:** Eric D. Ehler, Shane Edlund, Margaret Reynolds

**Affiliations:** ^1^ Department of Radiation Oncology University of Minnesota Minneapolis Minnesota USA

**Keywords:** 3D‐printed implants, adaptive radiotherapy, additive manufacturing, density override, megavoltage CT

## Abstract

Additively manufactured (3D‐printed) surgical reconstruction implants are increasingly encountered in head and neck oncology and may introduce density assignment challenges during radiotherapy treatment planning. This case report describes a clinically significant dosimetric discrepancy arising from the application of nominal bulk material density to an additively manufactured surgical reconstruction implant and to highlight workflow considerations for density assignment. A patient treated with helical tomotherapy following mandibular reconstruction with an additively manufactured titanium implant underwent kilovoltage computed tomography (kVCT) simulation. Saturation of Hounsfield units within the implant precluded reliable density estimation, necessitating a density override based on nominal bulk material properties. During treatment, megavoltage computed tomography (MVCT) imaging with adaptive dose recalculation was used to evaluate the dosimetric impact of this assumption. Replanning was performed using a revised density override informed by imaging‐based evaluation while maintaining institutional planning criteria. Adaptive dose recalculation demonstrated a localized calculated dose increase of approximately 7% within portions of the clinical and planning target volumes adjacent to the reconstruction implant. Replanning with a revised density override reduced unintended target dose heterogeneity while preserving target coverage. Nominal bulk material density may not accurately represent TPS‐relevant radiologic density for additively manufactured surgical reconstruction implants, and this discrepancy may not be apparent on kVCT imaging. Independent evaluation of density assumptions using alternative imaging modalities can identify clinically meaningful discrepancies and support patient‐protective replanning decisions.

## 1 LEARNING OBJECTIVES

After reviewing this case report, the reader should be able to:

- Recognize that nominal bulk material density may not accurately represent TPS‐relevant radiologic density for additively manufactured surgical reconstruction implants.
- Identify clinical scenarios in which kilovoltage CT imaging may be insufficient for density assignment due to Hounsfield unit saturation and metal‐related density uncertainty.
- Evaluate the role of adaptive dose recalculation using alternative imaging modalities as a clinical decision‐support tool when density assumptions are uncertain.

## 2 INTRODUCTION AND NARRATIVE

Mandibular reconstruction using patient‐specific implants has become increasingly common in the management of head and neck cancers following segmental mandibulectomy. Additive manufacturing, commonly referred to as 3D printing, allows reconstruction implants to be designed with complex geometries tailored to individual patients, improving surgical fit and functional outcomes. These mandibular reconstruction implants are frequently fabricated from titanium alloys, most commonly Ti‐6Al‐4V, due to their favorable mechanical properties and biocompatibility.^1^

In radiotherapy treatment planning, metallic implants present well‐recognized challenges related to imaging artifacts and dose calculation uncertainty.^2^ High‐density materials can cause beam hardening, streak artifacts, and saturation of Hounsfield units (HU) on kilovoltage computed tomography (kVCT), limiting the reliability of CT number–to–density conversion. In such situations, it is common clinical practice to apply a density override based on nominal bulk material properties provided by the implant manufacturer or published reference values.^3^ This approach implicitly assumes that the radiologic behavior of the implant is adequately represented by its specified material density.

For additively manufactured implants, however, this assumption may not always be valid. Unlike wrought or cast materials, additively manufactured titanium components can incorporate intentional internal porosity, lattice structures, surface roughness, and thin geometries^4^ that alter their effective radiologic density at the spatial scale relevant for radiation transport and dose calculation.^5^ When combined with partial‐volume effects and HU saturation on kVCT, these factors may lead to a mismatch between nominal material density and the implant's effective radiologic density used for dose calculation within the treatment planning system (TPS).

Helical tomotherapy systems (Radixact System, Accuray Inc., Sunnyvale, CA, USA) provide megavoltage computed tomography (MVCT) imaging that can be used not only for image guidance but also for dose recalculation through adaptive planning workflows.^6^ Since MVCT is more strongly Compton dominated than kVCT imaging, MVCT is generally less susceptible to metal artifacts and HU saturation, including material‐dependent photoelectric effects that can complicate kVCT interpretation. Although MVCT is not intended to provide ground‐truth material characterization in this work, its image‐value‐to‐density relationship is routinely calibrated and quality‐assured for clinical dose calculation. MVCT‐based adaptive recalculation can therefore serve as an independent evaluative check on planned dose distributions when kVCT‐based density assignment is uncertain.

This case is presented as an educational case report rather than a validation study, with the goal of illustrating a clinically relevant workflow failure mode and the decision‐making considerations that followed. We report a clinically significant potential dose deviation identified in a patient treated after mandibular reconstruction with an additively manufactured titanium implant. Saturation of kVCT HU values necessitated an initial density override based on nominal material properties. Subsequent MVCT‐based adaptive recalculation identified a localized increase in the calculated target dose, prompting replanning with a revised density assignment. This case highlights a potential failure mode associated with reliance on nominal bulk material density for additively manufactured mandibular reconstruction implants and demonstrates the role of MVCT‐guided adaptive evaluation in identifying and mitigating its clinical impact.

## 3 CASE DESCRIPTION

A patient with head and neck cancer underwent segmental mandibulectomy followed by reconstruction with an additively manufactured mandibular reconstruction implant. The implant was designed as a patient‐specific device based on preoperative imaging and surgical planning and was fabricated from a titanium alloy specified by the manufacturer as Ti‐6Al‐4V compliant with ASTM F136 (ELI Grade 23). The reconstruction implant consisted of a rigid, load‐bearing mandibular structure fixed with titanium hardware.

### 3.1 Simulation imaging

**FIGURE 1:**
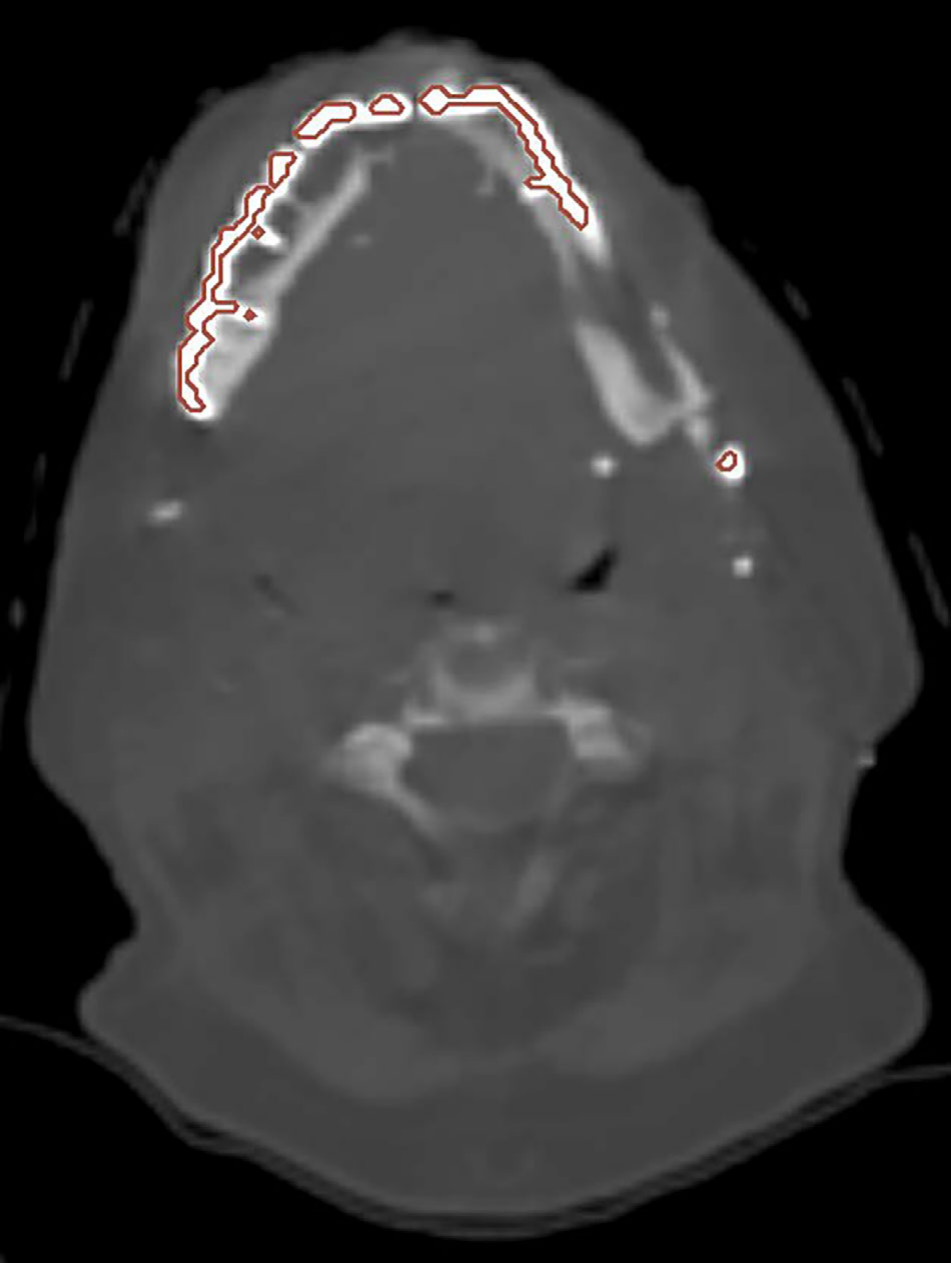
The kVCT simulation axial image demonstrating the additively manufactured mandibular reconstruction implant, outlined in maroon for clarity. The image was acquired with automatic metal artifact reduction applied and displayed using a window and level that span the full available HU range.

Simulation imaging was performed using the institutional radiotherapy kVCT simulation protocol with automatic application of a metal artifact reduction algorithm (Philips Big Bore/O‐MAR). Images were exported for treatment planning using 12‐bit CT image data. Automatic exposure control was enabled while tube potential was held constant according to the protocol. As shown in Figure 1, image appearance alone did not suggest a limitation in density assignment; however, the implant demonstrated heterogeneous HU values, with many voxels reaching the maximum CT number of 3071 HU, rendering the resulting HU values unreliable for density assignment.

### 3.2 Initial treatment planning

Treatment planning was performed using the vendor‐supplied helical tomotherapy TPS; dose was calculated using the system's convolution‐superposition algorithm. The treatment plan used 6 MV helical IMRT; the simulation CT slice thickness was 2 mm, and the dose was calculated using a 1.4 × 1.4 × 2.0 mm dose grid. The TPS used in this case maps CT image values to physical density for dose calculation; therefore, density values discussed in this report refer to physical density unless otherwise specified. Given the inability to derive a meaningful density from the kVCT dataset, a density override was applied to the implant volume. The override value was selected based on the nominal bulk material density of wrought Ti‐6Al‐4V titanium alloy^7^ (approximately 4.4 g/cm^3^). The treatment plan met institutional target coverage and organ‐at‐risk constraints and was approved for clinical delivery.

### 3.3 MVCT imaging and adaptive recalculation

After treatment initiation, routine MVCT imaging was acquired for image guidance. In contrast to the kVCT dataset, MVCT images did not exhibit HU saturation within the implant volume. Using the institution's quality‐assured MVCT image‐value‐to‐density calibration table, adaptive planning functionality within the TPS was employed to recalculate dose on the MVCT dataset using the original beam parameters. This calibration is verified at commissioning and monthly using commercial density phantom inserts, including lung and bone equivalent materials, water, and titanium. In‐house aluminum and stainless steel inserts are also included to represent high‐density materials encountered clinically, with a 50 HU tolerance relative to the commissioning baseline.

Adaptive dose recalculation, using the same dose grid resolution as the initial treatment plan, demonstrated a localized increase in calculated dose adjacent to and overlapping with the mandibular reconstruction implant. The magnitude of this increase was approximately 7% above the prescribed dose within portions of the clinical target volume (CTV) and planning target volume (PTV), with the dose‐difference DVH demonstrating a mean increase of 5.5% ± 1.7% and a maximum local increase of 9.0% within the implant density‐override structure. No dose differences of clinical concern were observed in other adjacent organs at risk. Review of the fraction MVCT registration and patient anatomy did not suggest setup deviation or gross anatomic change as the primary explanation for the localized dose difference, which was spatially concentrated near the implant and was considered most consistent with a density‐assignment effect. Adaptive recalculation of the revised plan was reviewed clinically and demonstrated no new concerning dose features.

### 3.4 Clinical decision and replanning

No fixed institutional dose‐volume threshold mandates replanning for this specific scenario. The decision was made on a case‐specific basis after physician and physics review of the localized calculated dose increase, its location in the reconstructed mandibular region, the potential risk of osteoradionecrosis, and the ability to reduce the calculated dose increase with a revised density assignment while maintaining treatment objectives. A revised density override informed by MVCT‐based density estimation was applied, and the plan was reoptimized while maintaining the original target and organ‐at‐risk objectives. The revised density override was informed by a review of representative MVCT voxel values within the implant contour. Voxels were selected from regions visually consistent with implant material while avoiding edge voxels, obvious partial‐volume regions, and residual artifacts. These values corresponded to apparent physical densities of approximately 2.5 to 2.8 g/cm^3^ on the institutional MVCT image‐value‐to‐density calibration, and a revised density override of 2.7 g/cm^3^ was selected for replanning. Effective density may vary substantially across additively manufactured implants depending on design and porosity, and therefore, case‐specific evaluation is warranted.

## 4 RESULTS

**FIGURE 2:**
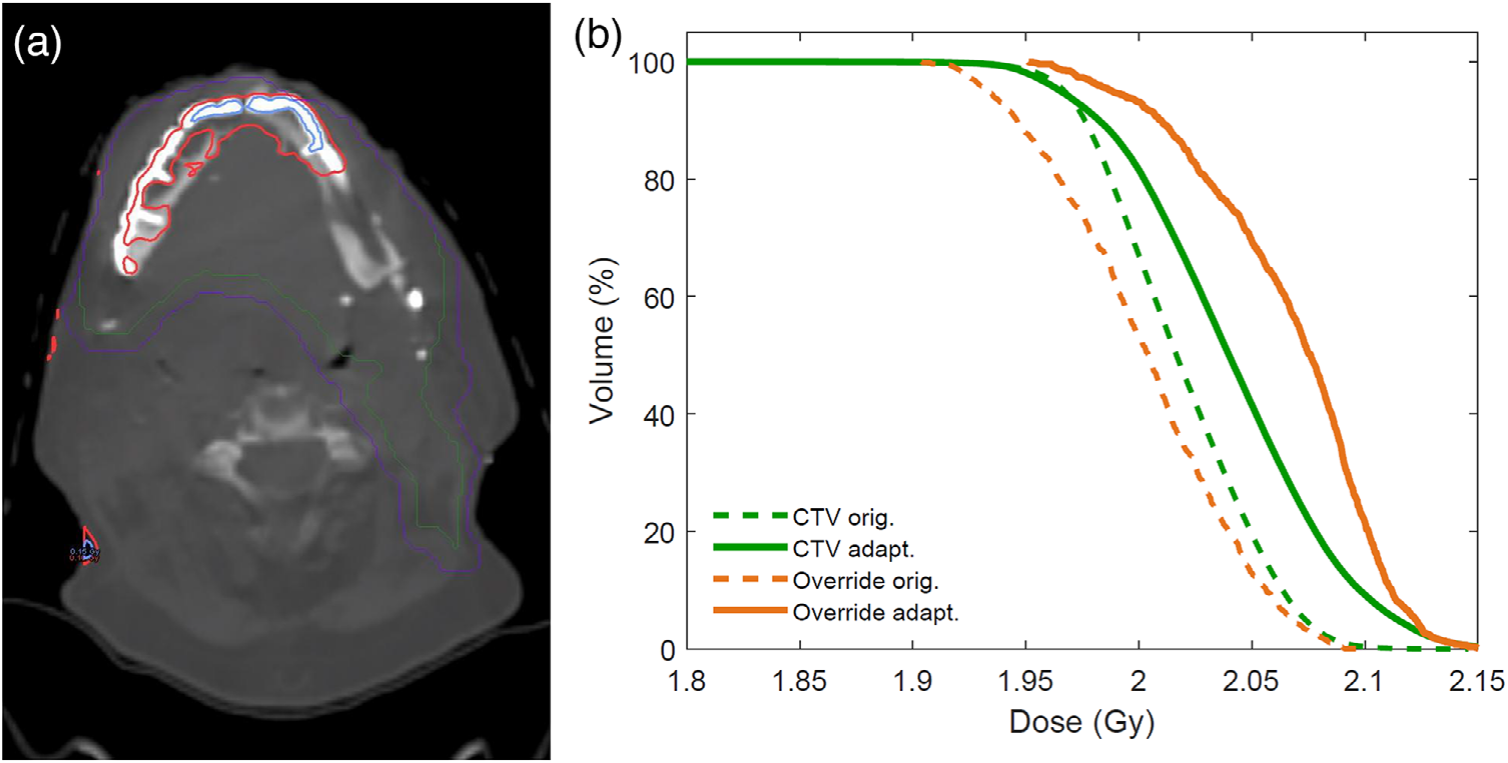
(a) Axial dose difference map demonstrating regions of increased dose under adaptive recalculation using MVCT‐derived density information relative to the original plan. Red and blue lines indicate 5% and 7.5% dose increases, respectively. (b) Dose–volume histograms for the CTV (green) and implant density‐override structure (orange), comparing the original plan (dashed) and adaptive recalculation (solid).

Adaptive recalculation of the original treatment plan using MVCT‐derived density information demonstrated a clinically meaningful localized dose increase adjacent to the mandibular reconstruction implant, including portions of the CTV and PTV. Dose increases were observed circumferentially along the implant–tissue interface, consistent with the rotational nature of helical delivery (Figure 2a). The dose increase was localized to the implant density‐override region, including portions overlapping the CTV and PTV. The corresponding dose–volume histograms (Figure 2b) demonstrate a rightward shift of the CTV and implant override structure under adaptive recalculation, reflecting the impact of the density assumption.

## 5 DISCUSSION

The central finding of this case is that nominal bulk material density may not accurately represent TPS‐relevant radiologic density for additively manufactured surgical reconstruction implants. While titanium alloys are generally considered well characterized in radiotherapy planning, this case demonstrates that assumptions derived from bulk material properties may not hold for patient‐specific, additively manufactured metallic devices, particularly when kVCT‐based density information is compromised.

Prior studies have shown that titanium fixation and reconstruction hardware can perturb local dose distributions through interface effects, scatter, and attenuation.^8^, ^9^ Metallic implants can also complicate radiotherapy planning through imaging artifacts and artifact management considerations.^10^ Existing studies provide important context for implant‐related dose perturbation and artifact management, but they do not determine how density should be assigned for a porous additively manufactured mandibular reconstruction implant. The present case adds to this literature by illustrating how reliance on nominal bulk material density for a patient‐specific additively manufactured implant can affect adaptive recalculation and clinical planning decisions.

Importantly, porosity in additively manufactured surgical reconstruction implants is an intentional design feature that promotes osseointegration and mechanical compatibility with bone; therefore, deviation from nominal bulk material density should be expected rather than considered anomalous. Additive manufacturing enables fabrication of complex metallic implants with geometries and microstructures that differ substantially from conventional wrought or machined components. Internal porosity, lattice reinforcement, surface roughness, and thin cross‐sections can alter effective radiologic density at the spatial scale relevant for radiation transport. Although standards such as ASTM F136 specify chemical composition and mechanical performance, they do not ensure radiologic equivalence to bulk material density in the context of additively manufactured, patient‐specific geometries.^11^

This case also illustrates that HU saturation is only one contributor to density‐assignment uncertainty for additively manufactured metallic implants. Implant porosity, geometry, and material‐dependent kVCT attenuation may also affect the interpretation of CT‐derived density values, particularly when conventional calibration materials do not reflect the implant design.

In this case, MVCT was not used as ground‐truth material characterization; rather, adaptive recalculation was used to evaluate the potential dosimetric consequences of an assumed bulk density when kVCT‐based density assignment was unreliable. Its role in this workflow was to identify a clinically relevant failure mode associated with density assumptions for additively manufactured implants. The availability of a quality‐assured MVCT image‐value‐to‐density relationship and adaptive dose recalculation enabled identification and mitigation of unintended target dose heterogeneity without additional imaging or intervention.

### 5.1 Clinical implications

As additively manufactured metallic implants become more prevalent, radiation oncology workflows must adapt accordingly. This case suggests that nominal bulk material density should be applied cautiously for additively manufactured implants, particularly when kVCT‐based density information is uncertain or incomplete. Independent imaging modalities and adaptive recalculation workflows can provide valuable insight into the dosimetric impact of density assumptions and support patient‐protective replanning decisions.

### 5.2 Limitations

This report is limited by its retrospective, single‐patient nature and the absence of direct physical measurement of implant density or independent dosimetric measurement for this patient‐specific implant geometry. MVCT was not treated as a ground truth for material characterization, and conclusions regarding implant density are necessarily inferential. This case report is intended to highlight a clinical workflow failure mode rather than establish frequency or generalizability. Additional cases and systematic phantom studies are needed to develop standardized density‐assignment approaches. Nevertheless, the magnitude and spatial localization of the observed dose discrepancy underscore the clinical relevance of the workflow considerations described.

### 5.3 Discussion questions

When density information from kVCT imaging is unreliable, what strategies are available at your institution to assess the dosimetric impact of density assumptions for metallic implants?

How should physicists balance nominal material specifications against imaging‐derived information when evaluating additively manufactured patient‐specific implants?

How should a clinical team decide whether to replan when adaptive recalculation suggests a possible hot spot near a metallic implant?

## 6 CONCLUSION

Nominal bulk material density may not reliably represent TPS‐relevant radiologic density for additively manufactured surgical reconstruction implants, particularly when kVCT HU saturation limits density assignment. Because these implants are intentionally engineered with internal porosity and complex geometries to promote osseointegration and mechanical compatibility, their effective radiologic density may differ substantially from bulk material specifications. In this case, reliance on nominal density resulted in a clinically meaningful localized target dose perturbation. MVCT‐guided adaptive dose recalculation identified the dosimetric consequences of this assumption and enabled mitigation through revised density assignment and replanning. As additive manufacturing continues to expand within radiation oncology, careful evaluation of density assumptions and incorporation of adaptive verification tools will be increasingly important to ensure accurate dose delivery.

## AUTHOR CONTRIBUTIONS

**Eric D. Ehler**: Conceptualization; methodology; investigation; formal analysis; visualization; writing—original draft, and writing—review and editing. **Shane Edlund**: Methodology; investigation; and writing—review and editing. **Margaret Reynolds**: Investigation; supervision; and writing—review and editing.

## CONFLICT OF INTEREST STATEMENT

The authors declare no conflicts of interest.

## ETHICS STATEMENT

This single‐patient case report describes a clinical observation identified during routine clinical care, with no intervention performed with prior research intent. The case information and images were prepared for publication in deidentified form. Based on institutional guidance, formal IRB review was not required for this type of case report.

## Data Availability

Data supporting this case report are not available for sharing.
